# Future disease burden due to the rise of emerging infectious disease secondary to climate change may be being under-estimated

**DOI:** 10.1080/21505594.2025.2501243

**Published:** 2025-05-11

**Authors:** Paul R. Hunter

**Affiliations:** The Norwich School of Medicine, University of East Anglia, Norwich, UK

**Keywords:** Climate change, emerging infectious disease, disease burden, immunity, virulence, severity

It is now widely accepted that climate change is increasing the risk of emerging infectious diseases, and that the impact of climate on infectious diseases will only become more severe over decades. The World Health Organisation has defined an emerging infectious disease as an infectious disease as “one that either has appeared and affected a population for the first time, or has existed previously but is rapidly spreading, either in terms of the number of people getting infected, or to new geographical areas” [1]. In recent decades, many such infectious diseases have been classified as emerging. Included in this list are HIV/AIDS, SARS, Covid-19, Monkeypox types 2 b and 1b, and Ebola.

Understandably, there is concern about whether climate change could increase the threat of emerging disease [2]. Climate change may lead to emerging diseases through multiple mechanisms [2]. These mechanisms may include, but are not limited to, changes in the geographic distribution of insect vectors or animal reservoirs, habitat destruction, or impacts of weather events on water and sanitation [2].

Several studies have attempted to model future changes in the incidence of infectious diseases. Modelling the future of dengue fever and malaria have been particularly rich topics in the literature [3]. However, these studies have covered other diseases, including cholera [4]. Most and perhaps all such studies have been concerned solely with future changes in the incidence of disease and not with disease burden.

Disease burden concerns not only the incidence of a disease but also its impact on society, which is usually measured as disability-adjusted years [5]. There appears to be an unstated assumption that the severity of the disease will remain roughly the same as the infection spreads from existing endemic areas into new populations. This assumption may lead to an underestimation of the impact of future emerging infections due to climate change.

It has long been argued that the spread of infection in previously naive populations is associated with increased mortality rates. Classic examples of this are the spread of black death through Europe (Figure 1) and the genocide associated with the spread of diseases in the New World [6]. Recent examples include both covid and zikavirus infections. During the first year of the covid pandemic mortality rates per infection were particularly high in subsequent years [7]. Zikavirus is endemic in many tropical countries [8], but its association with microcephaly, a devastating cause of brain damage in babies, was only identified when the infection started to spread in South America where it had not previously been seen [9].

**Figure 1.**
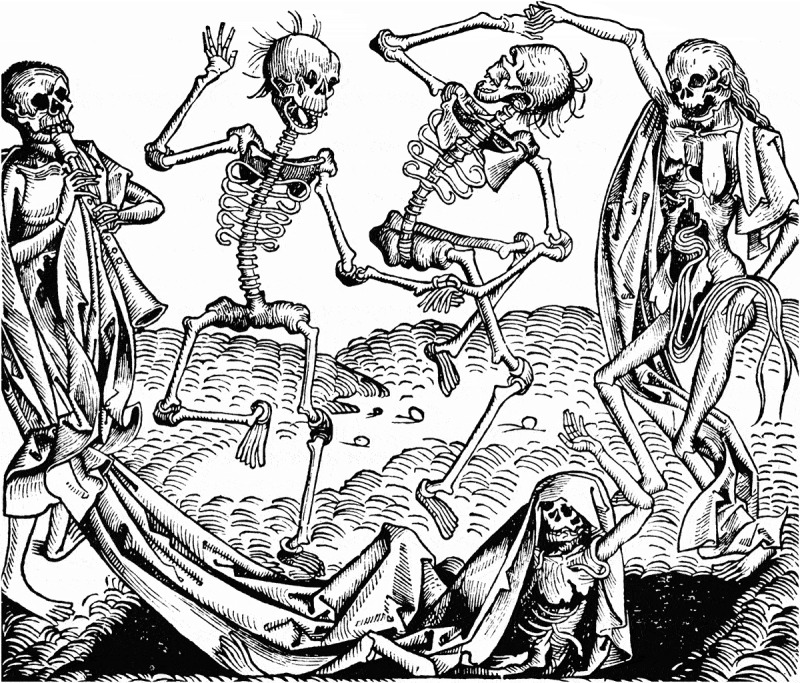
The dance of death (1493) by Michael Wolgemut, from the nuremberg chronicle of Hartmann Schedel.

Source: https://www.metmuseum.org/art/collection/search/390220.

Taken from https://commons.wikimedia.org/wiki/File:Nuremberg_chronicles_-_Dance_of_Death_(CCLXIIIIv).jpg Free from copyright

As new infections move into previously unexposed populations, deaths and disease burden could be substantially greater than would be estimated from extrapolation of the disease burden in populations where those infections are currently endemic. It seems to us that there are several possible mechanisms for why disease severity may be greater in previously unexposed populations.

Many infectious diseases cause more severe disease outcomes in older people and often cause relatively mild illnesses in younger children. This was most clearly seen in the data on mortality and hospitalization rates from covid infections [10]. For several endemic infections, it is the case that the majority of children will have experienced at least one infection by their fifth birthday as is the case with Respiratory syncytial virus [11]. Infection early in life may provide a degree of lifelong protection. For example, an influenza infection early in life will reduce the risk of a “medically attended infection” with the same subtype throughout life [12]. However, as an infection spreads into a previously unexposed population, the elderly will have as little prior immunity as preschool children and expect to suffer severe disease.

Zikavirus infection is a clear example of this. The initial zikavirus infection in adults is usually not severe [13] and is often asymptomatic [14]. However, if a woman is pregnant in her first trimester, there is a significant risk of severe brain damage to the foetus [15]. However, in populations where Zika is endemic, many women are likely to have had at least one infection by the time they have their first pregnancy, and so have immunity. As zika spreads in a previously unexposed population, adults will be at risk; therefore, one would expect ZIKV-associated microcephaly in newborns to be more frequent as found when the virus spreads into South America [9].

However, not all infections are severe in older people. Most causes of diarrheal disease tend to be more severe and cause higher mortality in young children than in older people [16]. Dengue virus is more complex. Initial dengue infections in children tend to be less severe than those in adults. However, dengue shock syndrome, associated with subsequent infections, tends to be more lethal in children.

Even initial infections in early infancy may be more severe in naïve populations. In immune populations, mothers can transfer protective immunity to their babies either transplacentally or while breastfeeding [17]. In naïve populations, mothers have little or no immunity, and so do not transfer such protection to their babies. This is the logic behind vaccines, such as whooping cough, being offered to pregnant women.

Not all differences in severity between populations can be explained by acquired immunity. Past pandemics have often increased evolutionary pressure that has led to an increased prevalence of mutations that confer resistance to otherwise lethal infectious diseases [18]. One example is that of the gene responsible for sickle cell disease. Despite causing sickle cell disease, the heterozygous state confers some protection against malaria and is found more commonly in populations where malaria is endemic [18]. As malaria moves into populations without this mutation, we could expect higher mortality rates.

So far, we have focused on the host factors that affect disease severity. merging infections are often more virulent early in the course of an epidemic [19]. While the observed reduction in disease severity over time may be due to factors such as improved health care, availability of vaccines and specific therapies, or increasing population immunity, some contribution may come from mutations towards lower virulence in the pathogen itself. This was observed with attenuated virulence in the omicron compared to the early variants of SARS-CoV-2 [20]. However, it should not be assumed that all emerging pathogens evolve to attenuate their virulence over time.

In conclusion, we should not assume that as infectious diseases increase their geographical distribution, disease severity in previously unexposed populations will be the same as that in populations where those diseases have been endemic for substantial time. Clearly, the outcome of emerging infections depends on many factors, including availability of adequate health services. Nevertheless, we need to plan, not just for increased incidence of infection as a result of climate change, but also for the possibility that those new infections could be more severe and have a more devastating impact on public health than currently expected based on experience in previously endemic settings.

## Data Availability

Data sharing is not applicable to this article, as no new data were created or analysed in this study.
